# Toward an Understanding of GSD5 (McArdle disease): How Do Individuals Learn to Live with the Metabolic Defect in Daily Life

**DOI:** 10.3233/JND-230027

**Published:** 2024-01-02

**Authors:** Walaa Karazi, Jacqueline Coppers, Daphne Maas, Edith Cup, Bart Bloemen, Nicole Voet, Jan T. Groothuis, Tomàs Pinós, Ramon Marti Seves, Ros Quinlivan, Nicoline Løkken, John Vissing, Salman Bhai, Andrew Wakelin, Stacey Reason, Nicol C. Voermans

**Affiliations:** a Department of Neurology, Donders Institute for Brain, Cognition and Behaviour, Radboud University Medical Center, Nijmegen, The Netherlands; b Department of Rehabilitation, Donders Institute for Brain, Cognition and Behaviour, Radboud University Medical Center, Nijmegen, The Netherlands; c Department for Health Evidence, Donders Institute for Brain, Cognition and Behaviour, Radboud University Medical Center, Nijmegen, The Netherlands; d Biomedical Network Research Centre on Rare Diseases (CIBERER), Instituto de Salud Carlos III, and Research Group on Neuromuscular and Mitochondrial Diseases, Vall d’Hebron Research Institute, Universitat Autònoma de Barcelona, Barcelona, Catalonia, Spain; e MRC Centre for Neuromuscular Diseases, UCL Institute of Neurology, National Hospital, London, UK; f Copenhagen Neuromuscular Center, Rigshospitalet, University of Copenhagen, Copenhagen, Denmark; g Department of Neurology, University of Texas Southwestern Medical Center, Dallas, TX, USA; h Neuromuscular Center, Institute for Exercise and Environmental Medicine, Texas Health Presbyterian, Dallas, TX, USA; i International Association for Muscle Glycogen Storage Disease, Torrance, CA, USA

**Keywords:** McArdle disease, GSD5, quality of life, adaptations, adjustments, physical activity, international survey

## Abstract

**Background:**

Glycogen storage disease type 5 (GSD) is an autosomal recessive inherited metabolic myopathy caused by a deficiency of the enzyme muscle glycogen phosphorylase. Individuals with GSD5 experience physical activity intolerance.

**Objective:**

This patient-led study aimed to capture the daily life experiences of GSD5, with a focus on adapting to and coping with their physical activity intolerance.

**Methods:**

An online survey was composed in close collaboration with patient organizations. It consisted of customized and validated questionnaires on demographics, general health and comorbidities, physical activity, psychosocial well-being and functioning, pain, fatigue and adapting to and coping with GSD5.

**Results:**

One hundred sixty-two participants (16 countries) participated. The majority, *n* = 86 (69%) were from the Netherlands, USA or UK. We observed a high rate of misdiagnosis prior to GSD5 diagnosis (49%), surprisingly a relatively high proportion had not been diagnosed by DNA testing which is the gold standard. Being diagnosed had a strong impact on emotional status, daily life activities and important life choices. A large proportion had not received any rehabilitation (41%) nor medical treatment (57%) before diagnosis. Engagement in vigorous and moderate physical activity was reduced. Health related quality of life was low, most likely related to low physical health. The median Fatigue Severity Score was 4.3, indicating moderate to severe fatigue. Participants themselves had found various ways to adapt to and cope with their disability. The adaptations concerned all aspect of their life, including household chores, social and physical activities, and work. In addition to lack of support, participants reported limited availability of information sources.

**Conclusion:**

Participants have provided guidance for newly diagnosed people, including the advice to accept one’s limited abilities and maintain an active lifestyle. We conclude that adequate counseling on ways of adapting and coping is expected to increase both health-related quality of life and physical activity.

## INTRODUCTION

GSD5 is a rare inherited metabolic myopathy caused by a deficiency of the enzyme muscle glycogen phosphorylase. To break down glycogen (stored form of glucose) for energy production, several enzymes including muscle glycogen phosphorylase (myophosphorylase) are needed. Individuals with GSD5 experience symptoms of muscle pain, fatigue and cramping within minutes of (strenuous) physical activity, and if activity continues at the same intensity they are at risk for acute rhabdomyolysis [1]. Life expectancy is normal, and thus far management is supportive in nature [2].

In the absence of a curative treatment, individuals have to learn to live with the metabolic defect caused by the condition. The degree to which individuals succeed in this is highly variable, probably related to the variation in age at diagnosis, personal coping styles, availability of emotional and physical guidance, participation in physical training, and peer support. Furthermore, comprehensive expertise for the rehabilitation of metabolic myopathies is not widely available. A recent study showed that most individuals with GSD5 have received incorrect explanations of their symptoms, and that misdiagnosis plays an important part in delaying the implementation of appropriate medical advice and management [3]. Furthermore, Health related quality of life (HRQOL) is severely reduced in individuals with GSD5 compared with normative data for non-affected subjects [3, 4].

For individuals that are afforded the opportunity to learn how to cope with the metabolic defect, physical activity is easier, enabling them to become more fit, which in turn results in fewer limitations in daily life activities [5]. In addition, adequate coping is likely to prevent recurrent episodes of acute rhabdomyolysis that often lead to hospital admission and the attendant risk of renal failure [5]. This calls for increased awareness of the importance of adequate counseling on how to be physically active in daily life and for physical training by an experienced physical therapist. The International Association for Muscle Glycogen Storage Disease (IamGSD) plays an important role by providing detailed online, open-access information, organizing walking courses, and led the composition of an international evidence-based Clinical Practice Guideline (CPG) [6]. However, not all individuals and caregivers are familiar with this organization.

Studies on how individuals with GSD5 learn to adapt to and cope with the metabolic defect have not been performed. This survey therefore aimed to explore how individuals with GSD5 adapt to and cope with their physical activity intolerance in daily life and sports, and learn to be physically active in a safe and satisfying way. The results of this survey will be a starting point for developing a patient led coaching and training program on implementing physical activity in a satisfying and safe way.

## METHODS

### Composition of the survey

The survey was composed in close collaboration with the board members of IamGSD. Furthermore, we invited eight individuals from the Dutch patients registration at the Radboudumc (age range 17–64 years, 5 females) with GSD5 to participate in a Focus Group. We explained our aims and discussed the topic of the survey. This discussion contributed to the composition of the customized questions and selection of the validated questionnaires, which resulted in a comprehensive first draft of the survey. This draft version was pilot-tested and checked for readability and clarity by three individuals with GSD 5 s of this Focus Group and by two members of the IamGSD board. Finally, feedback from two clinicians (N.C. Voermans and S. Bhai) was taken into consideration. The final version of the survey was created in Castor EDC, and all data were stored in Castor EDC. Reminders to participate were placed on the Facebook group and by email every two weeks and reminders to complete the survey were sent every two weeks to the participants who had already started participation. *Ethical approval:* The local medical ethical committee of the Radboudumc approved this study (METC Arnhem- Nijmegen 2022-13636).

### Participants and recruitment

Potential participants were identified via hospital-based registries at the expertise centers of Radboudumc, the Netherlands and Rigshospitatet, Denmark; via IAMGSD; the international McArdle Facebook group; and national GSD5 patient organizations (Dutch, Danish, French, Spanish and German).

The Dutch individuals received a secured email and letter at their home address and were invited to contact the research team for participation. The Danish individuals received the invitation in a secure email in their digital mailbox (e-Boks). After receiving informed consent, a link to the online survey was sent by email, and each participant was able to fill out the questionnaire. The patient organization IamGSD announced the study on its website and Facebook group. National patient organizations announced the study on their websites or informed their members by email. Individuals were invited to contact the research team for participation. Informed consent was obtained digitally before starting the survey, in line with the consent procedure previously applied by IamGSD [7].

Inclusion criteria included: GSD5 diagnosed by a medical specialist; age≥12 years; and ability to read and understand English or Dutch. Participant acquisition and data collection took place between June and August 2022. Participant enrollment is depicted in Fig. 1A.

**Fig. 1A:**
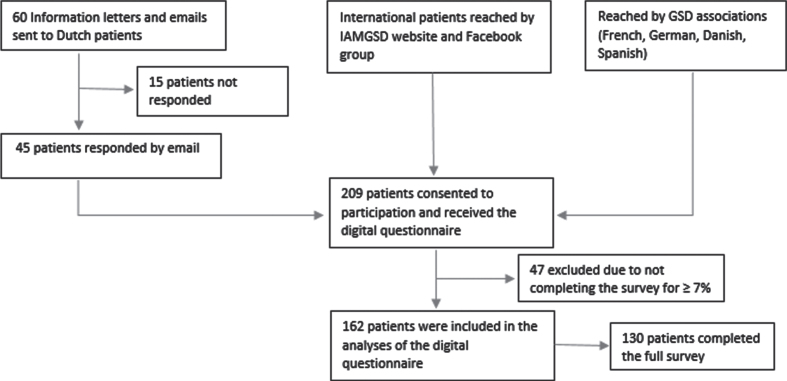
Participant enrollment.

### Questionnaires

The survey consisted of customized and existing validated questionnaires. The customized questions were on demographics (age, age at diagnoses, sex, education, country and social participation), general GSD5 related questions (misdiagnosis, symptoms, diagnosis, comorbidity, treatments) and coping (impact life choices, emotions, advice given, barriers and adjustments in various aspects). Both open and closed questions were included and clarification on answers was asked. Questions from the following validated questionnaires: International Physical Activity (IPAQ), Pediatric quality of life (PedsQL), Fatigue Severity Scale (FSS) and Adaptation to Disability Scale (ADS-R) were included. The full survey is available as supplemental data [30, 31].

The IPAQ short version that was used, consists of 4 generic items. The purpose of the questionnaires is to provide common instruments that can be used to obtain internationally comparable data on health–related physical activity. The score is expressed as MET-min per week and the Freedson cut point categorizations were used for data interpretations [11–13].

The PedsQL is a modular approach to measuring health-related quality of life in children, adolescents and adults. We used the short form (SF15) of version 4.0. It contains 4 multidimensional scales: physical functioning, emotional functioning, social functioning and school/work/studies functioning [14, 15]. Items of the PedsQL questionnaire were transformed to reversed scores from the 5 point Likert scale from 0 (never) to 5 (almost always). The reversed scores form linearly transformed scores to a 0–100 scale and higher scores indicate a better HRQOL [14].

FSS is a method of evaluating the impact of fatigue and contains 10 items. With the FSS scores, a mean between 1–7 can be calculated. A score of≥4 generally indicates moderate to severe fatigue [8]. Correlation of FSS with visual analog scale was r = 0.68; *p* < 0.001; vitality scores, were inversely correlated with FSS [8, 9].

The ADS-R is a 12-item measure of disability acceptance based on the four value changes (enlarging the scope of values, containing the effects of the disability, subordinating the physique, and transforming comparative-status values to asset values) [10]. The score can be calculated to a 32-item scale [26].

Answers to open-ended questions were summarized, discussed and interpreted by the study group.

### Statistical analysis

Descriptive statistics were used to characterize the data. We assessed frequencies, minimum and maximum, medians, means in case of normally distributed data and standard deviation (SD). All analyses were performed using IBM SPSS Statistics (software version 25. Armonk, NY: IBM Corp).

## RESULTS

### Demographics

In total, 162 participants were included in this study, of whom 130 completed the full survey (completion rate of 80%).

The median age was 52 years. The median age at diagnosis was 28 years, the median age of first symptoms was 5 years. Just above half, *n* = 69 (59%) were college graduates or higher, and *n* = 44 (39%) were currently working. While *n* = 30 (27%) indicated restrictions in their work or school, only *n* = 11 (10%) changed their job due to illness or had their employer change their work environment. Figure 1B provides an overview of the geographical origin of the participants. The majority, *n* = 86 (69%) were from the Netherlands, USA or UK. Detailed demographic characteristics are presented in supplementary data 2.

**Fig. 1B:**
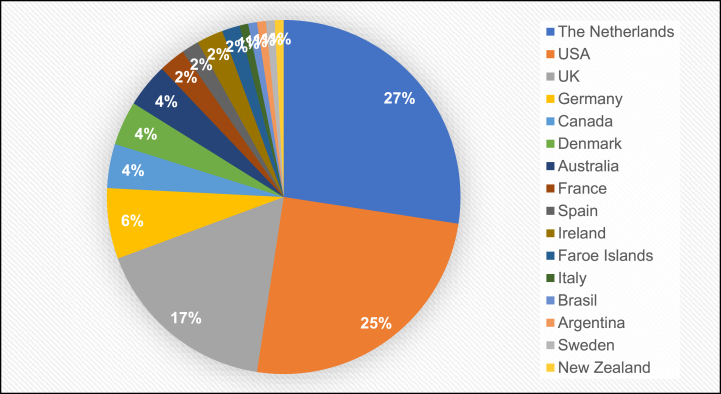
Country of origin of participants.

### Diagnosis

All participants indicated they had been diagnosed with GSD5 by a neurologist or other medical specialist and provided the name of the hospital they were diagnosed at. The most common method of diagnosis was muscle biopsy, *n* = 56 (45%), *n* = 32 (26%) by both biopsy and genetic testing, surprisingly only *n* = 25 (20%) by genetic testing alone and *n* = 10 (8%) reported diagnosis by other means (for example (non-)ischemic forearm test).

Nearly half of the participants, *n* = 58 (49%) reported they had been misdiagnosed prior to a GSD5 diagnosis.

When asked how they felt, *n* = 80 (62%)were relieved at diagnosis, while *n* = 5 were surprised, *n* = 15 concerned, *n* = 7 happy, *n* = 9 angry and *n* = 14 felt another emotion. Of the *n* = 80 participants that indicated they felt relieved, *n* = 61 clarified that the relief resulted from finally knowing that there was a medical reason for their symptoms and that it was not due to being lazy or unwilling. In addition, confirmation that GSD5 is not a life threatening condition contributed to feelings of relief and happiness. Lack of available knowledge and absence of a cure for GSD5 caused concern. Not being understood or taken seriously by doctors and other peers led to anger. The reported impact of GSD5 on life was variable: *n* = 25 (24%) reported an impact on all aspects of their life and *n* = 46 (44%) on multiple aspects of their life; *n* = 29 (28%) reported only little impact and *n* = 4 (3%) no impact at all. For the majority, diagnosis of GSD5 has affected important life choices as outlined in Fig. 2A. The effect on life choices was heightened following diagnosis related to work, living conditions and parenting, while it decreased for education (school).

**Fig. 2A:**
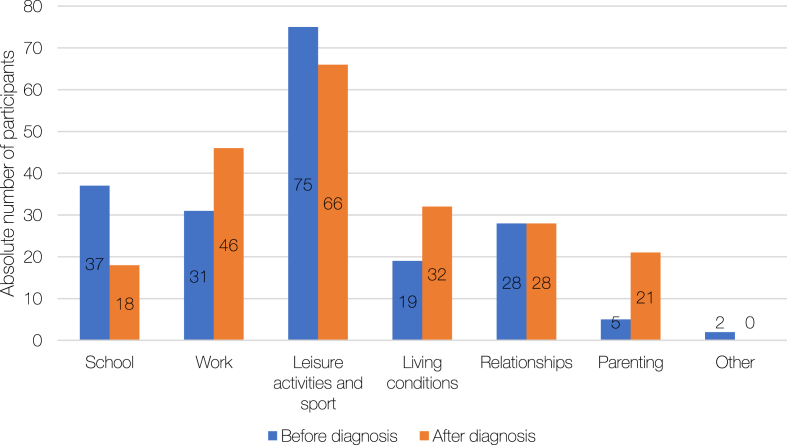
Effect of glycogen storage disease 5 (GSD5) diagnosis on life choices. Total respondents: *n* = 133.

### Symptoms

Figure 2B provides a summary of the reported symptoms before diagnosis and at the time of the study. Most participants reported difficulties in physical activity, *n* = 103 (77%), both before being diagnosed and at the time of the survey. Sixty-four (48%) reported being able to achieve second wind before diagnosis, while *n* = 99 (74%) reported being able to currently achieve second wind. Muscle fatigue was reported by *n* = 89 (67%) before diagnosis and by *n* = 94 (71%) at the time of the survey. Other symptoms were analyzed, and the most frequently reported symptoms included myalgia, pain, bladder cramps, excessive tiredness/chronic fatigue, being sick and/or feeling nauseous and the biomarker of a raised baseline serum creatine kinase (CK).

**Fig. 2B:**
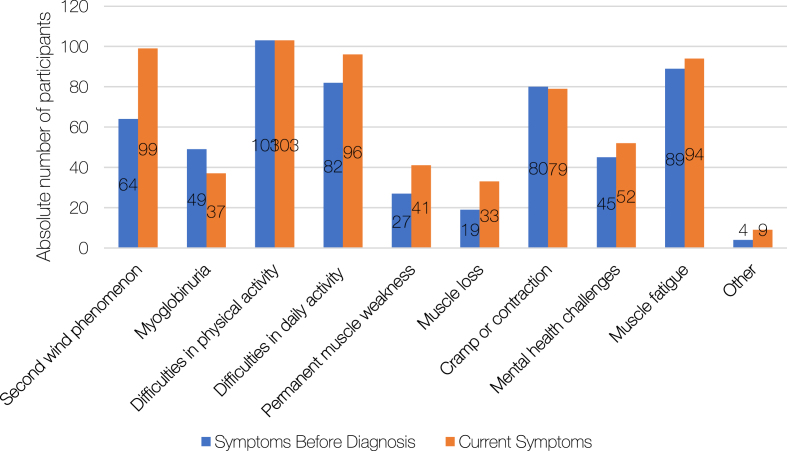
Symptoms of *glycogen storage disease 5 (GSD5)* before and after diagnosis. Total respondents: *n* = 133.

### Comorbidities

Most frequent comorbidities reported by participants included hypertension, varicose veins and ‘sleep problems’. A detailed list of comorbidities is available in supplementary data 3 and it is important to note that they may or may not be associated with GSD5.

### Treatments

Table 2 outlines treatments, both previous and current, that participants sought for GSD5 symptoms. Fifty (43%) participants indicated they have previously had physical therapy, whereas *n* = 18 (15%) were currently undergoing physical therapy. Psychological support (*n* = 23; 20%), occupational therapy (*n* = 20; 17%) and nutrition counselling (*n* = 25; 22%) were frequently reported as a previous treatment. Forty-eight (41%) participants stated that they had not received any previous rehabilitation management and *n* = 71 (64%) were not undergoing any rehabilitation at the time of the study. As for medication, *n* = 66 (57%) had not received a previous medical treatment for GSD 5 related symptoms. Medications for pain relief (over the counter + prescription) had previously been used by *n* = 42 (36%) participants, with *n* = 50 (43%) indicating they currently took medication for pain management. Muscle relaxants, antidepressants and anti-anxiety medications were also reported to have been used for GSD5 related symptoms.

### Physical Activity (PA)

The results of the IPAQ are included in Table 1. Fifty (47%) participants did not engage in any vigorous physical activities (such as heavy lifting) during the last 7 days, 31 (29%) did not engage in any moderate PA (such as bicycling) and 14 (13%) reported that they did not walk for at least 10 minutes during the last 7 days. The median time participants spent engaged in PA per day was as follows: vigorous PA (10 minutes), moderate PA (30 minutes), walking (38 minutes), and sitting (6 hours per week day). In comparison with the normalized value of the calculated MET-min per week (Freedson categories) and total PA min per week this sample scored low for PA [12, 13].

**Table 1:** Results of the validated questionnaires

Validated questionnaire	N	MEAN (SD)	MEDIAN	INTERVAL [MIN;MAX]	RANGE SCORES (MIN-MAX)
IPAQ (Total MET-min/wk)	105	2696.0 (3126.6)	1420	[0.0;16104]	Low, moderate or high
PedsQL	108	55.5 (16.4)	53.3	[10–95]	[0–100]
PsychoSocial components		69.0 (18.8)	70.0	[15–100]
Physical components		28.4 (21.1)	25.0	[0–100]
FSS	107	36.0 (12.1)/ 4.0	39.0/ 4.3	[0.00;54.0]	[9–63/1–7]
ADS-R	107	2.3 (1.3)	2.3	[1.00;3.25]	[1.0–4.0]

**Table 2:** Previous and current ongoing treatments for *glycogen storage disease 5 (GSD5)* related symptoms

	Previous Ongoing Treatments	Currently undergoing treatments
N	116	116
*Medication:*
Pain relief over the encounter	27 (23.3%)	33 (28.5%)
Pain relief prescription	15 (12.9%)	17 (14.7%)
ACE inhibitors	–	1 (0.9%)
Diuretics	1 (0.9%)	2 (1.7%)
Cardiovascular drugs	1 (0.9%)	4 (3.5%)
Insulin or antidiabetics	–	1 (0.9%)
Muscle relaxants	11 (9.5%)	9 (7.8%)
Psychoactive drugs	–	3 (2.6%)
Allopurinol	–	3 (2.6%)
Antidepressants	7 (6.0%)	11 (9.5%)
Antianxiety	1 (0.9%)	8 (6.9%)
Other	6 (5.2%)	12 (10.3%)
None	66 (56.9%)	57 (49.1%)
*Rehabilitation:*
Ketogenic diet	18 (15.5%)	10 (8.6%)
Nutrition counseling	25 (21.6%)	7 (6.0%)
Physical therapy	50 (43.1%)	18 (15.5%)
Occupational therapy	20 (17.2%)	3 (2.6%)
Psychological support	23 (19.8%)	6 (5.2%)
Other	2 (1.7%)	8 (6.9%)
None	48 (41.4%)	74 (63.8%)

### Quality of Life

The results of the PedSQL, are included in Table 1. The median for the total scale score was 53.3. The psychosocial health summary score (the sum of the items answered in the Emotional, Social, and Work/School functioning scales) for participants was 70 and the Physical Health summary score was 25. The low physical health score is notable, however psychosocial problems were cited; *n* = 36 (33%) stated that they sometimes worried about what will happen to them, *n* = 28 (26%) often and *n* = 21 (19%) almost always.

### Pain and Fatigue

Participants were asked how often they experience GSD5 related muscle pain lasting for more than 5 minutes after they stop activity, and how much this GSD5 related muscle pain interferes with their enjoyment of life. Over the course of 3 months, *n* = 26 (24%) experienced GSD5 related muscle pain once per 3 months, *n* = 24 (22%) once per month, *n* = 27 (25%) once per week, *n* = 18 (17%) once per day, *n* = 13 (12%) more than once per day. Nine participants (8%) stated that it did not interfere with their enjoyment of life at all, *n* = 26 (24%) a little bit, *n* = 34 (31%) moderately, *n* = 31 (29%) quite a bit and *n* = 6 (6%) extremely.

Participants were also asked to describe in their own words the sensations they feel within the first minute or two of activity/exercise after they push too hard. Their comments are summarized in supplementary data 4. Burning, pain and cramping sensations were most frequently mentioned..

Table 1 presents the results of the validated questionnaires. The median for the FSS, is 4.3. Thus, this sample is considered to be moderate to severely fatigued. Also, participants were asked to describe which number correlates the best to their general fatigue, 0 being worst and 10 being normal (median: 5.0).

### Adaptation and coping

The median for the ADS-R is 2.33. Table 3 presents the domains of the ADS-R and the scores. For approximately 66% of participants, GSD5 affects the aspects of life which they care most about. In this study, the median score was, 75, which reflects a moderate level of disability acceptance [26].

**Table 3:** Domains of the Adaptation to Disability Scale-Revised. *Disabled or not-disabled as subjectively defined and calculated to 4 point Likert scale (1 = strongly disagree-4: strongly agree)

Domain /Facet	Mean (SD)	Median	% ‘disabled’*
Feel like an adequate person	3.2 (1.8)	3.0	33%
Make good in life	3.4 (1.7)	3.0	33%
Many things able to do	3.4 (1.7)	3.0	33%
Affects most important aspects of life	2.6 (1.9)	3.0	66%
Disability foremost in mind all the time	2.4 (1.9)	2.0	33%
Sad and upset	2.0 (1.9)	2.0	33%
Good physical appearance and physical ability most important in life	2.2 (1.9)	2.0	33%
Physical wholeness and appearance makes the person	2.0 (1.8)	2.0	33%
Good and whole body for good mind	2.0 (1.8)	2.0	33%
Little to offer other people	1.7 (1.7)	2.0	33%
Disability worst thing	1.4 (1.6)	1.0	0%
Closed areas of life because of disability	1.6 (1.7)	1.0	33%

To better understand adaptations and coping, we explored difficulties in daily life (Table 4). The participants stated that the greatest barriers to managing daily life with GSD5 included: delayed diagnosis (*n* = 28), rarity of disease and insufficient knowledge of the disease by health care providers (*n* = 25), muscle pain (*n* = 23), lack of understanding by others (*n* = 19) and no prospect of a cure (*n* = 7). The most challenging aspect of living with GSD5 was not being able to plan activities because of unstable motor performances (*n* = 35), having muscle cramps very easily (*n* = 20), dealing with fear (*n* = 11), managing the risk of recurrent rhabdomyolysis (*n* = 10), knowing what level of pain is acceptable (*n* = 9) and dealing with bad moments/time (*n* = 8) (other *n* = 11). Open-ended responses regarding adjustments were analyzed and are summarized in Table 5. Forty-five (39%) participants mentioned that they did not participate in sports at all and *n* = 28 (24%) mentioned keeping their own pace and resting between repetitive activities as the most important adjustments. At school and work, participants made adjustments by avoiding heavy lifting and finding alternatives such as laptop trolleys. For household chores, participants mentioned electric alternatives to avoid heavy lifting and overdoing. Twenty-eight (24%) mentioned the importance of taking frequent breaks and going slow. Thirty-four participants (29%) shared that the type of social activity and the people they go out with is important; making adjustments is easier amongst friends/relatives that know about and understand GSD5. Seventeen participants (15%) did not need to make any adjustments in daily life. Participants shared that communicating personal limits and not being embarrassed about this are important adaptation strategies.

**Table 4:** Categorized results of the open questions, the mentioned difficulties in their daily life

Category	Key terms	Examples	N=
Acidification	Acidification, contractures	‘getting quick to acidification’	2
ADL challenges	Shower, brushing hair, putting socks/clothes on, chewing, writing, open jar, cooking, groceries, changing diapers, adult intimacy, brushing teeth, vacuuming, carrying, bathing child, household chores	‘Easy things like drying my hair of lifting my younger brother makes my arms feel dead’	56
Avoiding physical activities	Exercise, swimming, sports,	‘Exercise is out of the question’	4
Climbing stairs	Climbing stairs	‘I have to rest if I am walking more than two stairs.’	26
Walking	Walking	‘I can’t walk far.	45
Consequences for pushing too hard	I do too much/ too long, I push too hard, I don’t stop, carried on against the signals, forced to go over my limit. Examples consequences: pain, blocked/ swollen muscles, dark urine contractures, rhabdomyolysis	‘If I go over my limit I am punished almost immediately. Too many activities during the day, means a bad or no sleep at night due to pain/cramps’	6
Fatigue/ exhaustion	Less energy, (generally) tired, exhaustion, (muscle) fatigue	‘Quick onset fatigue, has become more frequent and severe.’	51
Recognizing pushing too hard or not	Can’t recognize, can’t make out/distinguish.	‘I can’t distinguish the signs of crossing a boundary from getting to second wind’	4
Getting to second wind		‘I struggle with SW. Doesn’t seem to kick in like It should.’	9
Starting any activity	Startup of.., starting activities	‘Actually, with every start-up of an activity and every repetitive movement that requires strength, I experience difficulties.’	5
Limits Social contacts/ activities/ sports/ work		‘The fact that you can’t just go for a bike ride or a hike, without planning in advance means that you are definitely limited in terms of social contacts/activities’	4
Mental challenging	Afraid, fear, accepting, mental issues, not being understood, not noticeable, barrier	‘any emotional or anxiety episodes will put me to bed for a couple days’	12
Pain (during activity)	Pain, not able to continue, hurt, have to stop, mild pain, pain during activity, extreme pain, muscle pain	‘I am getting soon to a point where I experience heavy muscle pain after exhausting activities. Sometimes longer than a day’	26
Stop or slowing down to allow recovery	Lots of rest breaks, slowly start, need a break, have to frequently stop, can’t continue without stopping,	‘I have to start the day very slowly.’ ‘I have to stop to allow muscles to recover.’	16
Variable day to day	Variable/varies, it depends on, from moment to moment,	‘Sometimes it works, other times it doesn’t. ’	9

**Table 5:** Categorizes results of the open questions, the mentioned adjustments per category

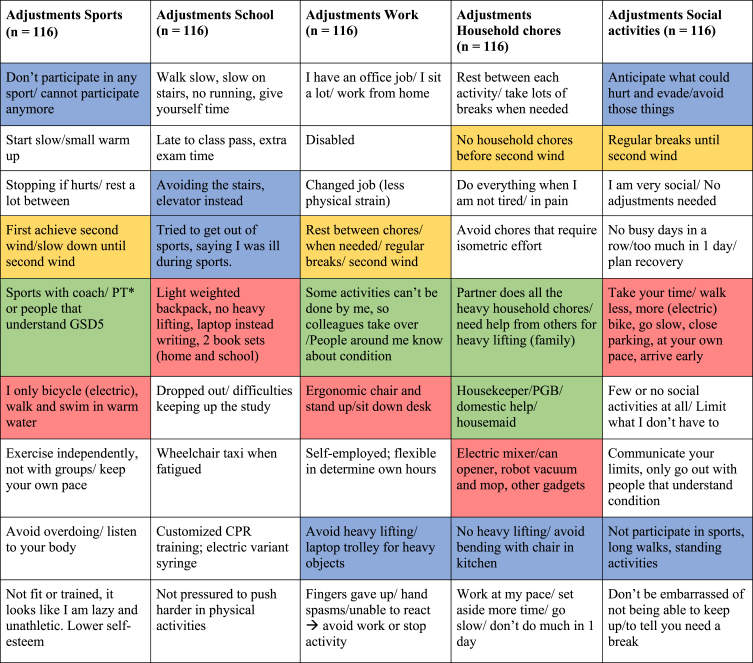

Red: Using assistive devices, Green: using help from others, Blue: avoiding certain activities, Yellow: using second wind. *PT = Personal trainer.

When asked who or what helped the most with day-to-day management, participants indicated the following: help from parents, siblings and other family members [*n* = 36 (29%)]; information provided by IamGSD [*n* = 14 (11%)]; rehabilitation [*n* = 12 (10%)], physical therapy and psychological support [*n* = 12 (10%)]; the IamGSD walking course [*n* = 7 (6%)]; peer support [*n* = 7 (6%)]; friends [*n* = 6 (5%)]; neurologist [*n* = 5 (4%)]; other means [*n* = 24 (20%)].

Furthermore, participants were asked to state if they had any tricks for coping with GSD5. From the 94 responses, only *n* = 9 participants stated they do not have any tricks. Just over half (*n* = 56) stated they pretend to do something (e.g. getting a call, tying shoelaces, gazing, checking phone, searching something) in order to be able to stop and rest till the pain and/or fatigue is gone. A few (*n* = 8) stated they pretend to have other physical problems, like circulation problems, backache, pain in knee, or blame their physical limitations on age. A minority (*n* = 11) stated that they had used a lot of tricks in the past but now felt they can be open about having GSD5. They shared that they have accepted their physical limitations, and have learnt how to adjust, or that they did not want to make excuses any longer. One participant stated that receiving the diagnosis had contributed to stop pretending. Improvements in lifestyle, like vitamins and fasting were also mentioned as ‘tricks’.

Finally, Table 6 outlines the advice participants would give to individuals newly diagnosed with GSD5. The majority [*n* = 75 (71%)] made the recommendations to accept your abilities and to maintain an active lifestyle.

**Table 6:** The advices participants would give to other newly diagnosed individuals

Advice	N
Accept your abilities and maintain an active lifestyle	75
Use information from patient organization IAMGSD	56
Find support from family members	51
Visit to a specialized neurologist	40
Find psychological support	28
Seek support from colleagues	27
Start physical therapy	26
Find support at school	21
Try the ketogenic diet	20
Start a rehabilitation program (multidisciplinary)	19
Eating carbohydrates before exercise	16
Participate in walking course by IAMGSD	15
Talk to a sports coach	12
Start occupational therapy	8
Other	11

## DISCUSSION

This international online survey sought to understand how individuals diagnosed with GSD5 adapt to and cope with their physical activity intolerance in daily life and sports, and how they learn to be physically active in a safe and satisfying way. As is the case with rare conditions diagnosis was most often delayed, the median participant age (52 years), age at diagnosis was 28 years while age of first symptoms was 5 years). This finding together with other clinical features reported in this study are in line with recently reported studies [3, 5, 7, 16–18]. Dutch and Danish individuals were recruited through the hospital-based registries, and only genetically confirmed participants were included in the survey data.

We were surprised to find a number of participants had been diagnosed by either muscle biopsy alone or by another means such as a forearm exercise test alone. Genetic testing is now readily available and is the ‘gold standard’ for diagnosis, it is possible that those participants who had not received a genetic diagnosis had been diagnosed many years ago before such testing was readily available. We decided not to exclude any participants on the basis of their diagnostic investigations since they had a clinical diagnosis of GSD5 made by a neurologist, although we accept this is not ideal and may possibly include some false positive diagnoses. However, the aim of this study was to understand the impact of having a diagnosis of GSD5 on the individual, and all participants included in this study had been given such a diagnosis.

The validated questionnaires showed a reduced HRQOL (with a severely reduced physical health score), moderate to severe fatigue, pain interfering with daily life activities, and reduced physical activity. The customized questionnaire provided insight into the adaptation and coping strategies participants use throughout their life. These strategies encompassed all aspect of life, including household chores, social and physical activities, and work. In addition to lack of support, participants reported limited availability of sources of information. Participants provided guidance for newly diagnosed people, including the advice to accept one’s limited abilities and maintain an active lifestyle.

In line with previous studies, we observed frequent (49%) misdiagnosis [7]. The diagnostic delay and misdiagnosis is in part related to the varying ways individuals described their symptoms (burning sensation, cramping sensation and muscle pain). In addition, muscle fatigue/pain are ubiquitous symptoms for many more common conditions. As GSD5 is considered a rare disease, it remains difficult to recognize and diagnose by physicians lacking in experience of this rare disorder. The most commonly reported explanations for symptoms given by health professionals were being lazy or unfit. Previous studies have shown that the long diagnostic delay can seriously affect QoL [2, 7]. Self-image may be impacted from being repeatedly told one is lazy and unmotivated, resulting in long-lasting feelings of insecurity and inferiority [27]. Our study adds that 71% report that being diagnosed had an impact on multiple or all aspects of their life. Overall, being diagnosed resulted in a feeling of relief.

The results of the validated questionnaires on QoL, fatigue, pain and physical activity are largely in line with previous findings. Previous research has collected population HRQOL measures for the PedsQL 4.0 generic core scales in physically healthy children and other diseases like asthma that can be used to compare scores across populations. The mean that is found for healthy adolescents is 79.45 (SD 16.40) [14]. This indicates a difference between the HRQOL for GSD5 participants and healthy population. Similar to other studies, QoL is severely impaired in GSD5 [7, 19]. Given that the mean age of participants in our study was 52 another validated questionnaire on the QoL (such as 12-item or 36-item Short Form Health Survey (SF-v12/SFv-36) would have been better suited. However, we had expected that more children would participate and therefore had chosen the PedsQL. Nevertheless, the use of the PedsQL contributes to the generalizability of results since three different validated questionnaires resulted in similar findings. Furthermore, the HRQOL scores of another study [19] were classified by sex, age, BMI category and other correlations. We observed that scores obtained in the vitality domain were higher in participants clinically diagnosed more years ago, which highlights the importance of an early diagnosis. Overall, the mean PedsQL score recorded in our (and their SF-36) sample was lower for the physical domains than for the mental domains. For 60% of the participants, GSD5 related muscle pain interferes moderately or quite a bit with their enjoyment of life, which may explain the low scores in the psychosocial components of the PedsQL. For a comparison of the FSS from this sample, the FSS score for Lyme disease is 4.8 and 6.1 for fibromyalgia, while for the healthy population it is considered to be 2.3 [8, 30]. For a comparison of the ADS-R score, the ADS-R mean score for individuals with epilepsy is 80 with a 32-item scale (range 32–128) [25].

Moreover, for *n* = 35 the most difficult or challenging aspect of living with GSD5 is not being able to plan activities because motor skills are not stable. Adaptation of planning for (social) activities is necessary. This sample scored a low physical activity score, in line with previous studies [5, 20–22] It is well known that individuals with GSD5 can benefit from aerobic training and that maintaining an active lifestyle has a positive impact [5, 22]. Remarkably, improvements in parameters are observed already after several weeks of training. Nevertheless, physical training support is unfortunately not widely available to individuals with GSD5.

The current study provides insight into the difficulties individuals with GSD5 face and the adaptations they make to improve their physical activity tolerance. We showed that many participants avoid participation in sports (39%), in particular, vigorous activity (47%). This is likely related to the lack of professional support and limited knowledge of health professionals on how best to advise on safe modes of training. Many participants reported that they had to discover these adaptations themselves. They eventually learn to keep their own pace during activity and take a rest between repetitive strenuous activities.

The ability to recognize the second wind phenomenon increased amongst participants from *n* = 64 to *n* = 99 following diagnosis. It is likely that a diagnosis was accompanied by an increase in understanding of the participants in the metabolic mechanisms underlying their condition. A large proportion of participants (41%) had not received any rehabilitation advice, which may explain why although most individuals recognized the second wind phenomenon many still had no tools or strategies to manage activities in daily life.

Many participants report the use of analgesic drugs, which again may reflect the lack of appropriate supportive exercise advice. The use of analgesics can potentially dampen the ‘normal’ GSD5 muscle signals during exercise that warn the individual to rest and thus increase the risk of rhabdomyolysis. There is a lack of consensus regarding nutritional and pharmacologic treatment to improve symptoms and studies to evaluate the role of ketogenic diet in improving exercise tolerance in GSD5 suggest that this may be a promising nutritional strategy [23, 24, 28]. Furthermore, obesity secondary to a sedentary life-style can pre-dispose to other more serious co-morbidities and carrying extra weight can exacerbate exercise intolerance. Nutritional advice on maintaining a healthy lifestyle is therefore important for people with GSD5. Our survey showed that only *n* = 25 respondents received nutrition counseling. More individuals with GSD5 recommended eating either carbohydrates before exercise or a ketogenic diet to manage GSD5. IamGSD conducted an informal survey that described improvement in symptoms and physical activity tolerance after using the ketogenic diet. This led to other studies investigating the effects of a modified ketogenic diet in individuals with GSD5, *but yet no long term efficacy and safety are proven*. The international clinical practice guidelines supports clinicians in multiple disciplines [2].

Participants reported use of various coping strategies to avoid embarrassment and stigma in social situations, for example, finding an excuse to stop walking such as looking at their phone or pretending to tie a shoelace. They reported that teaching yourself these coping skills does not happen deliberately but individuals find themselves doing it. Participants provided their own guidance for newly diagnosed people with GSD5, including the advice to accept one’s limited abilities, maintain an active lifestyle, and not to feel embarrassed by their condition.

This study has a number of limitation. The first limitation has been discussed above: the use of the PedsQL as the quality of life questionnaire for all participants. We had deliberately chosen this questionnaire in anticipation of the participation of children. In hindside, however, we could have better used the 12-item or 36-item Short Form Health Survey. This would have allowed for better comparison with the quality of life in other neuromuscular disorders. Another limitation is ascertainment bias. The survey was developed for a rare disease, with the intent to include as many participants as possible with a diagnosis of GSDV. To achieve this aim we used social media as recruitment method, and were not able to verify reported clinical diagnoses of GSD5 from medical records. To limit this bias, we asked participants to include details of the hospital and doctor looking after them, which was provided by 48% of the respondents. Of course, it would have been better to include the genetic test results, since this serves as a gold standard for the diagnosis of GSD5 V. The patients knowledge of these test results was deemed low and therefore not included. To improve this, we are a strong advocate of providing the genetic information at the moment of diagnosis in any rare disease. Genetic confirmation is also likely to be essential for future participation in clinical trials. Furthermore, the forearm exercise test, which is much less invasive than muscle biopsy, has a very high sensibility and specificity for diagnosis of GSD5 [29]. Both methods can contribute to a more limited number of invasive muscle biopsies. This study highlighted a distinct lack of appropriate diagnostic methods used in GSDV by clinicians.

We wanted to include participants from different countries but non-English and/or non-Dutch speaking countries were effectively excluded from this survey as it was not made available in different languages. Furthermore, a number of participants who did not complete the survey. This is likely related to the length of the survey. Moreover, recall bias is possible due to the retrospective design of the study. Finally, as the focus of this study was to explore the participant perspective of having a diagnosis of GSDV we did not include a control group.

Nevertheless, this study is the first to specifically explore adaptation to and coping with physical activity intolerance in people diagnosed with GSDV. Another strength is the extensive patient participation in composition and execution of the survey.

To conclude, our findings suggest that the diagnostic methods used in McArdle disease fell below recognized international standards and that appropriate medical management including rehabilitation was lacking. Physician education on GSDV would be an important step forward in improving medical management since HRQOL can improve when individuals are diagnosed at a younger age, enabling them to become physically active and adapt favorably to living with their condition. Availability of various resources is crucial for education and newly diagnosed individuals can learn from experienced individuals who have already adapted to and coped with restrictions in everyday activities. In addition, clinicians are likely to benefit from the experiences shared by individuals with GSD5 and advocacy groups, IamGSD has made strong advancements in patient advocacy, however not all individuals are aware of these resources. We aim to continue working towards a better understanding and counseling of individuals with GSD5 worldwide. Future studies should focus on methods to improve physical activity (structured exercise program) and improving the availability of information on adaptation and coping. This is expected to improve the health and well-being of individuals with GSD5.

## FUNDING

We are grateful to the Stichting McArdle and Stofwisselkracht for funding this research project. We acknowledge IAMGSD for providing very valuable suggestions to this project.

## Supplementary Material

Supplementary Material
