# Supplementary material for: Toward an Understanding of GSD5 (McArdle disease): How Do Individuals Learn to Live with the Metabolic Defect in Daily Life
Source: J Neuromuscul Dis. 2024 Jan 2;11(1):103–16. doi: 10.3233/JND-230027 (PMC10789332; doi:10.3233/JND-230027)
Supplement: Supplementary Material [file jnd-11-jnd230027-s001.docx]

**Supplemental Data**

**Supplementary 1**: For the full survey you can visit the following PDF: [Questionnaire study among McArdle 2.0 PDF.pdf](file:///H:\Spierziekten%20Centrum%20Radboudumc\Stage_W%20Karazi_McArdle\PaperPart1Co-authors\Questionnaire%20study%20among%20McArdle%202.0%20PDF.pdf)


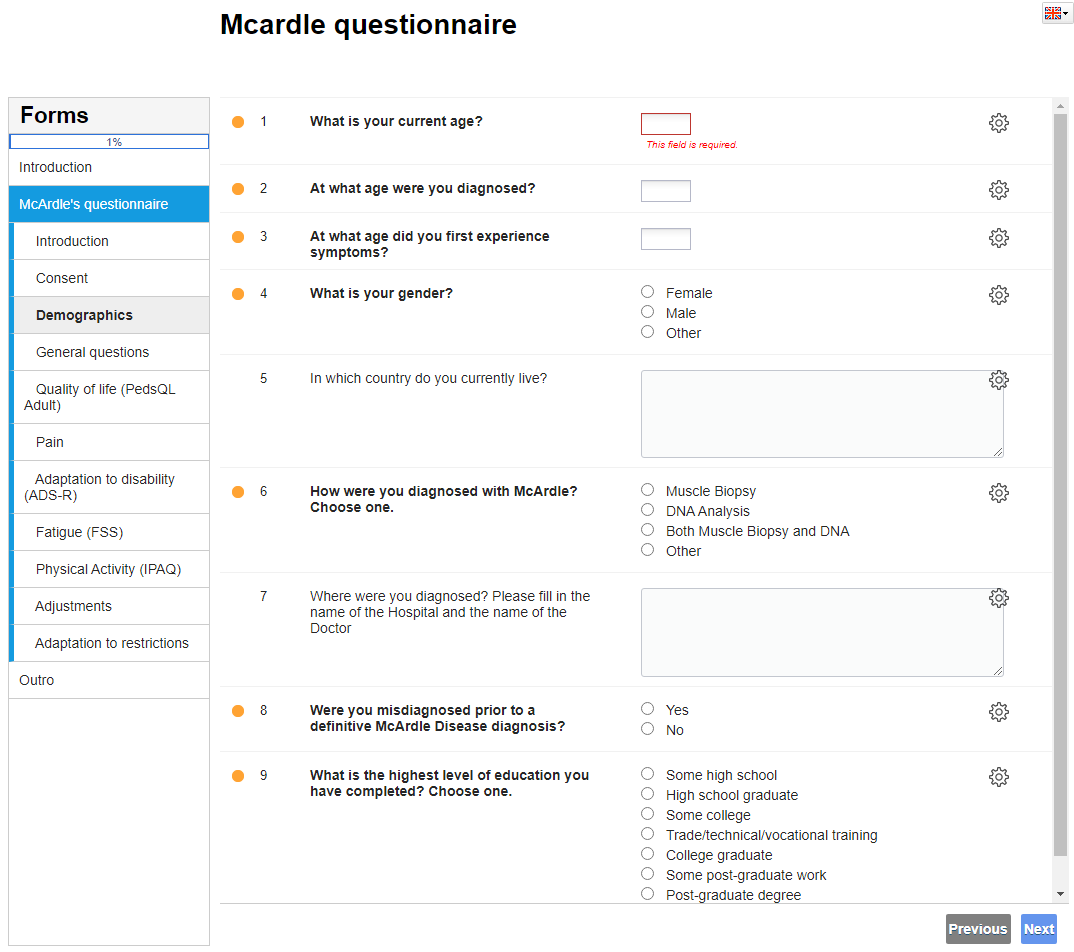


**Supplementary 2:** Demographic and Social Data

|  | **N (percentages)** | **Mean (SD)** | **Median** | **[Min-Max]** |
| --- | --- | --- | --- | --- |
| **Sex** | Female: 74 (59.7%)  Male: 50 (40.3%)  Total: 124 | - | - | - |
| **Age current** | 128 | 49.7 (18.0) | 52 | [12-87] |
| **Age at diagnoses** | 128 | 31.5 (16.3) | 28 | [4-76] |
| **Age first symptoms** | 128 | 7.7 (9.1) | 5 | [0-72] |
| **Highest education**  **level** | Some years of high school  High school graduate  Some college  Trade/technical/vocational training  College graduate  Some post-graduate work  Post-graduate degree  I prefer not to answer | | 3 (2.6%)  10 (8.6%)  14 (12.1%)  20 (17.2%)  29 (25.0%)  10 (8.6%)  26 (22.4%)  4 (3.5%)  Total: 116 | |
| **Social participation** | Currently at school  Currently working  Changed job due to illness  Employer changed environment due to illness  Restrictions in work/school/sport  I prefer not to answer | | 8 (7.2%)  44 (39.6%)  7 (6.3%)  4 (3.6%)  30 (27.0%)  18 (16.2%)  Total: 111 | |

**Supplementary 3**: Comorbidities for glycogen storage disease 5 (GSD5)

**Supplementary 4:** Description of the sensations felt within the first minute or two of activity after they push too hard.

| **Category** | **Key terms** | **Examples** |
| --- | --- | --- |
| **Burning** | Burning sensation, burning pain, muscle burning | I feel like there’s a burning in my quads to my calves and feet. |
| **Cramping** | Cramped, stiffed/ stiffness, stiff muscles, cramping sensation, muscle cramp, cramping in legs, cramps for the next 2 days. | Cramp in legs, almost as though I can’t physically put one foot in front of the other. I have to stop until the cramping goes away, then I can continue. |
| **Raised heart rate** | Fast/rapid/racing heartbeat, high pulse, heart palpitations | rapidly raised heart rate |
| **Nauseas** | Want to vomit, nauseous | I get very nauseous |
| **Pain** | Muscle pain, extreme pain, painful stiff, severe pain, terrible pain, sharp pain, chest pain, violent pain, | Sometimes severe muscle pain that goes away after a while but sometimes lasts for several days. |
| **Shortness breath** | Heavy breathing, breathless, shortness of breath | Very short of breath, like the world is caving in. |
| **Heaviness** | Heavy legs, lead in arms/legs, legs feel like tree trunks, | I feel like there is 200 pounds on my thighs and if I keep going they will lock. |
| **Emotional** | Fear, embarrassed, anxiety, getting worried, afraid, pissed off | I feel pain at first, and then fear, that it was too much and I will have another kidney failure. |
| **Tiredness** | Tired, very tired, running out of energy, exhausted, abnormal fatigue | Like my muscles are tired  The feeling of running out of energy or exhaustion |
| **Weakness** | Muscle weakness, strengthless, no strength, like I could fall | I just feel weak. My body feels like jello but also immediately feels sore and tense. |
| **Tightness** | Tight feeling, legs begin to tighten, chest gets tight | Tightness in legs, muscles get sore, can’t continue without resting. Feels like the muscles or bones are being pulled apart/off. |
| **Can’t go further** | Have to stop, cannot move, moving on is hard, can’t lift my legs, block, can’t walk further, muscles freeze up, disfunction | Muscles that sometimes block.  Struggling to move further/ forward. |
| **Other** | Feeling sick, Very hot, sweaty, numbness, acidification, light headed, affected vision, dizziness | The numbness begins and I have to stop. My vision is sometimes affected, everything looks too bright, but flat. |
